# Atypical development of white matter microstructure of the corpus callosum in males with autism: a longitudinal investigation

**DOI:** 10.1186/s13229-015-0001-8

**Published:** 2015-03-11

**Authors:** Brittany G Travers, Do P M Tromp, Nagesh Adluru, Nicholas Lange, Dan Destiche, Chad Ennis, Jared A Nielsen, Alyson L Froehlich, Molly B D Prigge, P Thomas Fletcher, Jeffrey S Anderson, Brandon A Zielinski, Erin D Bigler, Janet E Lainhart, Andrew L Alexander

**Affiliations:** Occupational Therapy Program, Department of Kinesiology, University of Wisconsin-Madison, Madison, WI USA; Waisman Center, University of Wisconsin-Madison, 1500 Highland Avenue, Madison, WI 53705 USA; Department of Psychiatry, University of Wisconsin-Madison, Madison, WI USA; Department of Psychiatry, Harvard School of Medicine, Boston, MA USA; Neurostatistics Laboratory, McLean Hospital, Belmont, MA USA; Interdepartmental Program in Neuroscience, University of Utah, Salt Lake City, UT USA; Scientific Computing and Imaging Institute, University of Utah, Salt Lake City, UT USA; Department of Radiology, University of Utah, Salt Lake City, UT USA; Department of Pediatrics, University of Utah and Primary Children’s Medical Center, Salt Lake City, UT USA; School of Computing, University of Utah, Salt Lake City, UT USA; Department of Neurology, University of Utah, Salt Lake City, UT USA; Department of Psychology, Brigham Young University, Provo, UT USA; Neuroscience Center, Brigham Young University, Provo, UT 84602 USA; Department of Medical Physics, University of Wisconsin-Madison, Madison, WI USA

**Keywords:** Diffusion tensor imaging, Brain development, Developmental disorders, Childhood, Adolescence, Adulthood

## Abstract

**Background:**

The corpus callosum is the largest white matter structure in the brain, and it is the most consistently reported to be atypical in diffusion tensor imaging studies of autism spectrum disorder. In individuals with typical development, the corpus callosum is known to undergo a protracted development from childhood through young adulthood. However, no study has longitudinally examined the developmental trajectory of corpus callosum in autism past early childhood.

**Methods:**

The present study used a cohort sequential design over 9 years to examine age-related changes of the corpus callosum in 100 males with autism and 56 age-matched males with typical development from early childhood (when autism can first be reliably diagnosed) to mid-adulthood (after development of the corpus callosum has been completed) (3 to 41 years of age).

**Results:**

The group with autism demonstrated a different developmental trajectory of white matter microstructure in the anterior corpus callosum’s (genu and body) fractional anisotropy, which suggests atypical brain maturation in these regions in autism. When analyses were broken down by age group, atypical developmental trajectories were present only in the youngest participants (10 years of age and younger). Significant main effects for group were found in terms of decreased fractional anisotropy across all three subregions of the corpus callosum (genu, body, and splenium) and increased mean diffusivity, radial diffusivity, and axial diffusivity in the posterior corpus callosum.

**Conclusions:**

These longitudinal results suggest atypical early childhood development of the corpus callosum microstructure in autism that transitions into sustained group differences in adolescence and adulthood. This pattern of results provides longitudinal evidence consistent with a growing number of published studies and hypotheses regarding abnormal brain connectivity across the life span in autism.

**Electronic supplementary material:**

The online version of this article (doi:10.1186/s13229-015-0001-8) contains supplementary material, which is available to authorized users.

## Background

Autism spectrum disorder (ASD) is a genetically complex neurodevelopmental disorder that may be marked by atypical functional connectivity within and between particular neural networks and regions in the brain [1-4]. Atypical functional connectivity may implicate white matter, which contains bundles of axons that allow for fast and efficient neuronal communication [5]. The largest white matter tract is the corpus callosum, which facilitates interaction between the left and right hemispheres across multiple lobes. Studies in individuals with callosal agenesis or with a surgically severed corpus callosum suggest that the corpus callosum is involved in a diversity of functions but may be particularly involved in cognitive processes that require the integration of complex information (for a review, see [6]). Because persons with ASD have been shown to demonstrate difficulty with higher order or complex information processing tasks [7-9], the corpus callosum may be a key area of study in this disorder, and within-group corpus callosum differences may be able to help disentangle aspects of the clinical heterogeneity commonly observed in ASD.

Multiple sources of evidence suggest corpus callosum atypicalities in persons with ASD. The corpus callosum was one of the first brain structures observed to be abnormal in *in vivo* neuroimaging studies of autism [10-14]. Decreased mean size of the corpus callosum, especially the anterior aspects [15], is one of the most replicated structural imaging results of case-control studies of ASD (for a meta-analysis, see [16]). Functional connectivity measures have suggested decreased interhemispheric connectivity in young children, adolescents, and adults with ASD [2,17-19]. Because the corpus callosum is composed predominately of parallel myelinated axon bundles in primates [20], it is well suited for investigation using diffusion tensor imaging (DTI) [21] to examine the microstructural integrity of the corpus callosum in persons with ASD. DTI is based upon a Gaussian model of water diffusion in tissue and provides a description of white matter microstructure through the tensor measures, including the fractional anisotropy (FA), mean diffusivity (MD), radial diffusivity (RD), and axial diffusivity (AD). These DTI measures provide complimentary information about the microstructure of the corpus callosum. For example, MD is the average diffusivity over all directions and is inversely related to the density of cellular membranes. RD is defined as the shortest eigenvector of the diffusion tensor (perpendicular to the corpus callosum white matter fibers) and has been associated with myelin abnormalities [22,23]. AD is defined as the longest eigenvector of the diffusion tensor (parallel to the corpus callosum fibers) and is affected in axonal injury and sensitive to cytoskeletal features [24]. FA is a normalized measure of variance among the lengths of all three eigenvectors of the diffusion tensor. An FA close to zero represents isotropic diffusion with equally long eigenvectors, and an FA of one represents highly directional diffusion with maximal elongation of one eigenvector compared to the others.

FA has been the most common measure of white matter microstructure used in DTI studies of autism [25], but it is encouraged to supplement measures of FA with measures of MD, RD, and AD in order to more accurately characterize white matter microstructure [26]. White matter microstructure and corresponding DTI values can be affected by changes in myelination, axonal density, axonal degeneration, axonal packing, neuroinflammation, fiber crossing, fiber curving, or fiber branching. The specific pattern of group differences across FA, MD, RD, and AD may be more indicative of one type of white matter atypicality than another [24], suggesting the need to examine the pattern of results across all of the available metrics.

A number of recent studies have used DTI methods in cross-sectional examinations of white matter in ASD, and one of the most consistent findings is decreased FA and increased MD of the corpus callosum in ASD (for a review, see [25]). In addition, a subset of these studies has shown significantly increased RD but not AD in ASD [27-32]. Increased RD may reflect a thinner myelin sheath of the axons [33,22,23,34], which suggests that the axons in the white matter tracts of the corpus callosum are less myelinated in persons with ASD.

Despite the consistency of these results, there are a handful of studies that have found either increased FA or no group differences in corpus callosum microstructure [32,35-37]. Two of these studies investigated very young subjects (that is, 1.8-to-3.3-year-old children in [35] and 1.5-to-5.8-year-old children in [32]), and it is possible that this discrepancy in findings may be due to developmental effects very early in life. Indeed, in individuals with typical development, cross-sectional and longitudinal studies suggest that white matter tracts, including the corpus callosum, tend to increase in FA and decrease in MD from childhood into adulthood [38-43], and if there is a differing developmental trajectory of the white matter integrity in ASD, the detection of group differences may be more or less likely at particular ages.

Only longitudinal studies can satisfactorily examine developmental trajectories, and to date, only one longitudinal DTI study in ASD has been conducted in very young children. Wolff and colleagues [44] found that the high-risk infants who went on to develop ASD demonstrated significantly greater FA of the corpus callosum at 6 months of age, no difference in FA at 12 months of age, and significantly decreased FA at 24 months of age compared to high-risk infants who did not go on to develop ASD. These results suggest that young children who develop ASD may undergo distinct development of the corpus callosum microstructure during infancy. However, studies of older children and adults with ASD are needed. In typically developing individuals, the corpus callosum undergoes an uneven and prolonged developmental trajectory that likely continues through adolescence [39-43]. This protracted development of the corpus callosum through childhood and adolescence and indications of atypical corpus callosum structure in ASD demonstrate the need for a longitudinal examination of the corpus callosum in children, adolescents, and adults. Understanding the development of the corpus callosum in ASD may be critical, given that atypical development of this structure could have cascading effects on other aspects of brain development, including early cortical development and the formation of synchronous neuronal assemblies [45,46].

The present study extends the cross-sectional study of Alexander and colleagues [27] to longitudinally examine DTI age-related changes of the corpus callosum of 100 individuals with ASD and 56 age-matched individuals with typical development from early childhood (when ASDs can first be reliably diagnosed) to mid-adulthood (after development of the corpus callosum has been completed) (3.0 to 41.8 years old).

## Methods

### Design

**Figure 1:**
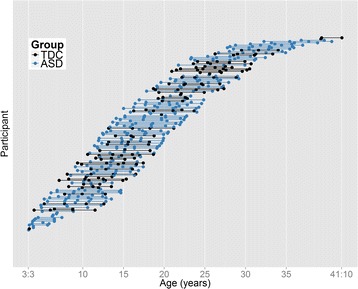
**Age at scan and number of scans for each participant in the longitudinal corpus callosum analyses.** ASD, autism spectrum disorder; TDC, typically developing controls.

The study employed a cohort sequential design (that is, an accelerated longitudinal design) [47], which simultaneously measured individual longitudinal changes in DTI measurements across multiple age cohorts. Each participant was imaged and clinical measures were collected one to four times over a 9-year period (see Figure 1). Fifty-six participants had four scans (41 ASD and 15 typically developing), 36 participants had three scans (22 ASD and 14 typically developing, 38 participants had two scans (20 ASD and 18 typically developing), and 26 participants had one scan (17 ASD and 9 typically developing). The average interscan interval was 2.6 years.

### Participants

The study protocol was approved by both the University of Utah and University of Wisconsin-Madison Institutional Review Boards. Consent was obtained for all participants, and for participants under the age of 18 years, both parental consent and participant assent were obtained. All TDC and the majority of the ASD participants were community ascertained. Participants for this study included 100 males with ASD and 56 males with typical development between the ages of 3 and 41. These participants were selected from participants in the broader longitudinal neuroimaging study (110 ASD and 78 TDC). Participants who met criteria for ASD or TDC at enrollment but who were found to be atypical at future time points (for example, one ASD participant developed a significant medical disorder affecting the brain; several TDC participants developed depression), participants who did not have an age match in the other group, or participants whose scans did not meet quality control checks (see ‘Imaging protocol’ below) were excluded. Time 1 data from a subset of these participants were reported in the study by Alexander and colleagues [27]. The participants with ASD were diagnosed based on Autism Diagnostic Interview-Revised [48], Autism Diagnostic Observation Scale (ADOS) [49], Diagnostic Statistical Manual-IV-TR [50], and ICD-10 criteria. Participants with ASD were included in the present study if they met criteria for a lifetime diagnosis of autistic disorder, Asperger’s syndrome, or pervasive developmental disorder not otherwise specified (89% met full criteria for autistic disorder). Exclusion criteria included medical causes of ASD (determined by patient history, physical exam, fragile-X testing, and karyotype), history of severe head injury, hypoxia-ischemia, seizure disorder, and other neurological disorders. ASD is thought to be a highly heterogeneous disorder [51], and the heterogeneity in the IQ domain of our ASD sample can be seen in the ranges of scores in Table 1. Typically developing control participants were confirmed as having typical development through history, the ADOS, IQ testing, and neuropsychological and standardized psychiatric assessment. All participants had English as their first language and were verbal at the time of testing. Forty-nine participants with ASD reported being on a centrally active medication (that is, stimulant, antidepressant, neuroleptic, atypical neuroleptic, or anti-anxiety medication) during at least one of the scans (38 at time 1, 31 at time 2, 19 at time 3, and 10 at time 4). See Table 1 for more detailed participant information.

**Table 1:** **Demographic characteristics of the longitudinal study sample and** ***P*** **-values for group comparisons**

	**ASD**	**TDC**	***P*** **-value**
Number of subjects	100	56	-
Scans per subject, mean (SD)	2.9 (1.1)	2.6 (1.1)	0.18
Scans at time 1	92	43	-
Scans at time 2	75	35	-
Scans at time 3	66	41	-
Scans at time 4	54	28	
Total number of scans	287	147	-
Age (years), mean (SD)	18.3 (8.5)	18.9 (7.8)	0.46
Age range	3.3 to 40.6	3.4 to 41.8	-
Interscan interval, mean (SD)	2.7 (0.4)	2.6 (0.5)	0.20
Interscan interval range	1.7 to 3.9	1.6 to 4.4	-
FSIQ^a^ mean (SD)	100.1 (17.6)	118.2 (13.2)	<0.001
FSIQ range	53 to 138	89 to 153	-
VIQ^a^ mean (SD)	96.3 (21.1)	114.7 (13.2)	<0.001
VIQ range	51 to 145	87 to 151	-
PIQ^a^ mean (SD)	102.6 (17.9)	116.3 (14.8)	<0.001
PIQ range	62 to 140	87 to 155	-

ASD, autism spectrum disorder; FSIQ, full-scale IQ; PIQ, performance IQ; TDC, typically developing controls; VIQ, verbal IQ. ^a^ Calculated as mean for each individual then averaged across each group.

### Assessments

#### IQ

Standard scores in verbal, nonverbal, and overall ability (referred to as verbal IQ [VIQ], performance IQ [PIQ], and full-scale IQ [FSIQ], respectively, despite differences in terminology across the tests) were assessed in the participants with ASD and the participants with typical development at each of the four time points. At time 1, participants completed the Differential Abilities Scales [52], Wechsler Intelligence Scale for Children (third edition) [53], or Wechsler Adult Intelligence Scale (third edition) [54] (depending on age and verbal ability). At time 2, participants completed the four subtests of the Wechsler Abbreviated Scale of Intelligence (WASI) [55]; at time 3, participants completed the two-subtest version of the WASI; and at time 4, participants completed the WASI if they had a previous IQ test, or participants completed the Differential Abilities Scales [52], Wechsler Intelligence Scale for Children (third edition) [53], or Wechsler Adult Intelligence Scale (third edition) [54] if they had not had a previous IQ test.

### Imaging protocol

Participants were recruited and scanned at the University of Utah. Image processing and analyses were conducted at the University of Wisconsin-Madison. A total of 346 scans were obtained from the participants (221 ASD scans and 125 TDC scans). Magnetic resonance images were acquired on a Siemens Trio 3.0 Tesla Scanner (Siemens, Munich, Germany) at the University of Utah. At all four time points, the DTI acquisition used a product single-shot, spin-echo, echo planar imaging (EPI) pulse sequence with diffusion weighting, performed with bipolar gradients with dual-echo refocusing to reduce eddy currents [56]. Parallel imaging with a geometric reduction factor of two was used to reduce image distortions from magnetic field inhomogeneities. For each slice, we obtained diffusion-weighted images in 12 non-collinear diffusion encoding directions with a *b* = 1,000 s/mm^2^ and a single *b* = 0 image. Sixty contiguous axial slices 2.5 mm thick were acquired over the cerebrum and cerebellum (matrix = 128 × 128; field of view [FOV] = 256 mm; resolution = 2 × 2 × 2.5 mm; repetition time [TR] = 7,000 ms; echo time [TE] = 84 ms at time 1 and 91 ms at times 2, 3, and 4; and pixel bandwidth = 1,346 Hz). The acquisition was repeated four times (52 volumes total) in 6.5 min. Thirty-two young participants with ASD received sedation for scanning at time 1, and fifteen of these same participants received sedation again at time 2. No sedation was used at times 3 or 4. Sedation, using a combination of remifentanil and propofol, followed a strict clinical protocol approved by the institutional review board by the University of Utah and performed by an onsite faculty anesthesiologist. No complications occurred. In several cases, rehearsal was used to practice lying in the scanner.

Between times 1 and 2, the scanner hardware (primarily the head coil: an 8-channel receive-only array coil at time 1 and a 12-channel receive-only array coil at times 2, 3, and 4) and software were upgraded (which accounted for the TE change described above). To account for systematic differences in the imaging measurements at time 1 versus times 2, 3, and 4 due to this upgrade, a linear regression ‘head coil’ variable (which also accommodated for pulse sequence differences) was created and included in all statistical models. To further ascertain that the present longitudinal results were not solely due to noise from this upgrade, all patterns of results were tested by running time 1 analyses only and then by dropping time 1 and using only times 2, 3, and 4 data (see Additional file 1, Tables S1 and S2).

### DTI image analysis

Diffusion-weighted images (DWI) were corrected for distortion, translation, and rotation from eddy currents and bulk head motion using an affine registration tools implemented in the fMRIB FSL software library [57]. To exclude regions of extreme intensities prior to processing, a mask was applied that excluded the upper 1% of the apparent diffusion coefficient, lower 1% of the average diffusion-weighted map, and lower 1% of the average b0 maps. The gradient orientation was corrected for rotation [58]. The DWI were smoothed in the axial plane using a 2D Gaussian kernel with 1.88-mm full width at half maximum (FWHM). Brain images were then skull-stripped. Next, the tensors were fit using a robust estimation algorithm (RESTORE) [59], implemented in Camino [60]. RESTORE has been shown to substantially reduce the effects of noise and physiologic artifacts from DTI maps by removing outliers in DWI measurements [61].

The eigenvalue maps (λ_1_, λ_2_, λ_3_) were computed from the estimated diffusion tensors. Maps of the MD (average of the eigenvalues), FA (the normalized standard deviation of the eigenvalues reflecting the relative degree of diffusion anisotropy), AD (λ_1_ the largest eigenvalue), and RD (λ_3_ diffusion perpendicular to the major eigenvector) were calculated. The units for MD, RD, and AD were square millimeter per second, scaled 10^−3^. Quality control checks were manually performed on the DTI images, looking for instances of slice intensity banding, FA hyperintensities, frontal lobe distortions, or blurring that could affect the analyses. Additionally, the white matter masks were checked to make sure that they were adequately covering the white matter in each brain scan. After quality control checks on the data, all but six ASD scans (98.6%) met the quality control standards.

To account for the effects of potential group differences in head motion during scanning, we computed the total motion index (TMI) for each participant as described in [62,63]. The TMI suggested that head motion did not differ between groups (*P* = 0.84) nor across ages (*P* = 0.48), but TMI was used as a covariate in all analyses to ascertain that group differences were not due to head motion. Additionally, to account for possible physiological noise, we computed the signal-to-noise ratio (SNR) for each region of the corpus callosum in each scan by taking the mean signal from all the volumes of the scan (b0 and the diffusion weighted volumes), dividing the signal by the noise in a voxel (noise estimated using the residual method described in the appendix of [61]), and then calculating the average SNR within each corpus callosum region of each scan. SNR of each region was used as a covariate in all analyses of that particular region.

A population-specific template was estimated iteratively, aligning all the subjects using affine and diffeomorphic diffusion tensor registration implemented in DTI-TK [64]. To segment the corpus callosum and its subregions (that is, genu, body, and splenium), the JHU ICBM-DTI-81 template FA [65] was registered to our population FA template using a diffeomorphic spatial normalization tool, ANTS [66], and the subregion labels were transferred to our template using nearest neighbor interpolation. Using a method similar to that described by Faria and colleagues [67], these regions of interest (ROIs) were then transferred to the native space of each participant using the inverse of the corresponding spatial transformations estimated during the population normalization. Median FA, MD, RD, and AD were calculated for each ROI. The median was selected as our measure of central tendency rather than the mean to protect against sensitivity to voxels with extreme values. To prevent partial volume effects, we generated histograms of the MD values for each scan and masked out any voxels that had MD two standard deviations above the mean for each scan. Then, corpus callosum white matter was further refined by masking out voxels with FA less than two standard deviations below the mean for each corpus callosum mask.

### Statistical analyses

To assess the reliability of the longitudinal DTI measurements, an intraclass correlation coefficient (ICC) was calculated for each statistical model by taking the within-individual variability in the mixed-effects model and dividing it by the sum of within and between variability [68]. Because development of the corpus callosum may not be fully captured by parametric methods, we took a semi-parametric approach that used penalized smoothing splines to examine age-related changes in DTI metrics. These are generalized additive mixed models [69-72] that also model repeated measurements per subject, are specially designed for our cohort-sequential sampling plan [73,74], and have been found to have empirical validity in different applications. These smoothing splines often bend to data more effectively than polynomials (while being penalized for each bend to prevent over-fitting), do not miss important nonlinear growth spurts or declines, and prevent fitted curve bias at the extremes of the data range. We used these smooth curves to examine the effects of age and diagnosis on the tensor coefficients, after taking into account head motion (TMI), head coil, and SNR. A centered FSIQ variable was initially included as a possible covariate, but it was in no case found to improve the fit significantly and therefore dropped. Finally, a number of more traditional general linear mixed-effects models were conducted to confirm the penalized spline models. Specifically, we divided the sample into three age groups (less than 10 years of age, 10 to 20 years of age, and more than 20 years of age) to examine the main effects of diagnosis and age on DTI metrics as well as age-by-group interactions within each of these age brackets.

## Results

### Longitudinal data reliability and consistency

To examine the reliability of the present DTI data, we calculated ICCs for each DTI measurement of each subregion (see Table 2). The average ICC was 83%, demonstrating good reliability (although AD of the body of the corpus callosum had much lower ICC than the rest of the measurements). Furthermore, to ascertain that our typically developing data was representative of the population at large, we compared this data to previous typically developing DTI studies with similar age ranges (that is, [42,43]) and found similar means, ranges, and growth curves for FA despite differences in data acquisition and analysis procedures. Therefore, these data demonstrated good within-individual consistency and consistency with previous reports.

**Table 2:** **Generalized additive mixed-effects models examining group and age-related changes in DTI metrics**

**Longitudinal DTI changes**										
	**Group**			**Age spline**			**Age X group spline**			
	**Mean**	**Estimate** ^**a**^	***P*** **-value**	**EDF**	***F***	***P*** **-value**	**EDF**	***F***	***P*** **-value**	**ICC**
Genu FA										
ASD and TDC	0.62	−3.12%	0.048	3.45	3.99	0.006	4.67	2.25	0.05	89.7%
ASD	0.61	-	-	4.31	2.11	0.075	-	-	-	
TDC	0.63	-	-	5.26	5.02	0.0002	-	-	-	
Body FA										
ASD and TDC	0.61	−2.40%	0.001	3.99	8.95	****	6.33	2.41	0.02	88.3%
ASD	0.61	-	-	6.59	3.46	0.002	-	-	-	
TDC	0.62	-	-	4.25	15.64	****	-	-	-	
Splenium FA										
ASD and TDC	0.69	−2.27%	0.04	5.91	9.66	****	1.00	0.19	0.66	81.1%
ASD	0.69	-	-	6.65	6.89	****	-	-	-	
TDC	0.7	-	-	3.67	11.54	****	-	-	-	
Genu MD										
ASD and TDC	0.76	1.63%	0.46	3.60	8.06	****	1.00	0.07	0.78	85.9%
ASD	0.76	-	-	3.04	5.20	0.002	-	-	-	
TDC	0.75	-	-	4.87	7.06	****	-	-	-	
Body MD										
ASD and TDC	0.79	1.90%	0.23	3.49	6.39	0.0002	1.30	0.06	0.86	82.4%
ASD	0.79	-	-	3.57	6.39	0.0002	-	-	-	
TDC	0.78	-	-	3.06	12.13	****	-	-	-	
Splenium MD										
ASD and TDC	0.77	1.89%	0.001	1.81	0.53	0.57	4.63	5.62	****	83.0%
ASD	0.78	-	-	4.58	8.25	****	-	-	-	
TDC	0.76	-	-	2.96	1.63	0.18	-	-	-	
Genu RD										
ASD and TDC	0.37	5.89%	0.23	3.52	3.91	0.006	3.38	1.50	0.21	87.8%
ASD	0.38	-	-	3.80	2.75	0.03	-	-	-	
TDC	0.36	-	-	4.87	5.89	0.00007	-	-	-	
Body RD										
ASD and TDC	0.4	5.33%	0.08	3.89	7.46	0.00001	4.93	1.47	0.20	91.7%
ASD	0.4	-	-	5.05	4.11	0.001	-	-	-	
TDC	0.38	-	-	4.00	15.00	****	-	-	-	
Splenium RD										
ASD and TDC	0.32	7.54%	0.01	4.72	7.92	****	1.00	0.70	0.40	86.6%
ASD	0.33	-	-	5.89	5.55	****	-	-	-	
TDC	0.31	-	-	3.34	8.66	****	-	-	-	
Genu AD										
ASD and TDC	1.38	−0.28%	0.60	3.99	5.53	0.0002	1.00	0.06	0.81	85.9%
ASD	1.38	-	-	3.10	6.10	0.0004	-	-	-	
TDC	1.39	-	-	1.87	8.58	0.0005	-	-	-	
Body AD										
	1.48	−0.005%	0.08	1.00	4.22	0.04	2.19	2.01	0.13	56.6%
	1.48	-	-	1.93	9.72	0.0001	-	-	-	
	1.48	-	-	2.66	3.25	0.03	-	-	-	
Splenium AD										
	1.55	0.35%	0.03	1.67	0.37	0.65	3.55	1.87	0.12	80.0%
	1.55	-	-	3.47	2.64	0.04	-	-	-	
	1.54	-	-	3.70	1.64	0.17	-	-	-	

^a^For the parametric group differences, the estimate is reported as the percent deviation in the ASD group compared to the TDC group with the corresponding *P*-value. For the age spline and the age-by-group splines, the estimated degrees of freedom (EDF), *F*-value, and *P*-values are reported. ****P-value <0.00005. AD, axial diffusivity; ASD, autism spectrum disorder; EDF, estimated degrees of freedom to assess smooth spline complexity; ICC, intra-class correlation coefficient; FA, fractional anisotropy; MD, mean diffusivity; RD, radial diffusivity; TDC, typically developing controls.

### Longitudinal corpus callosum trajectory models

**Figure 2:**
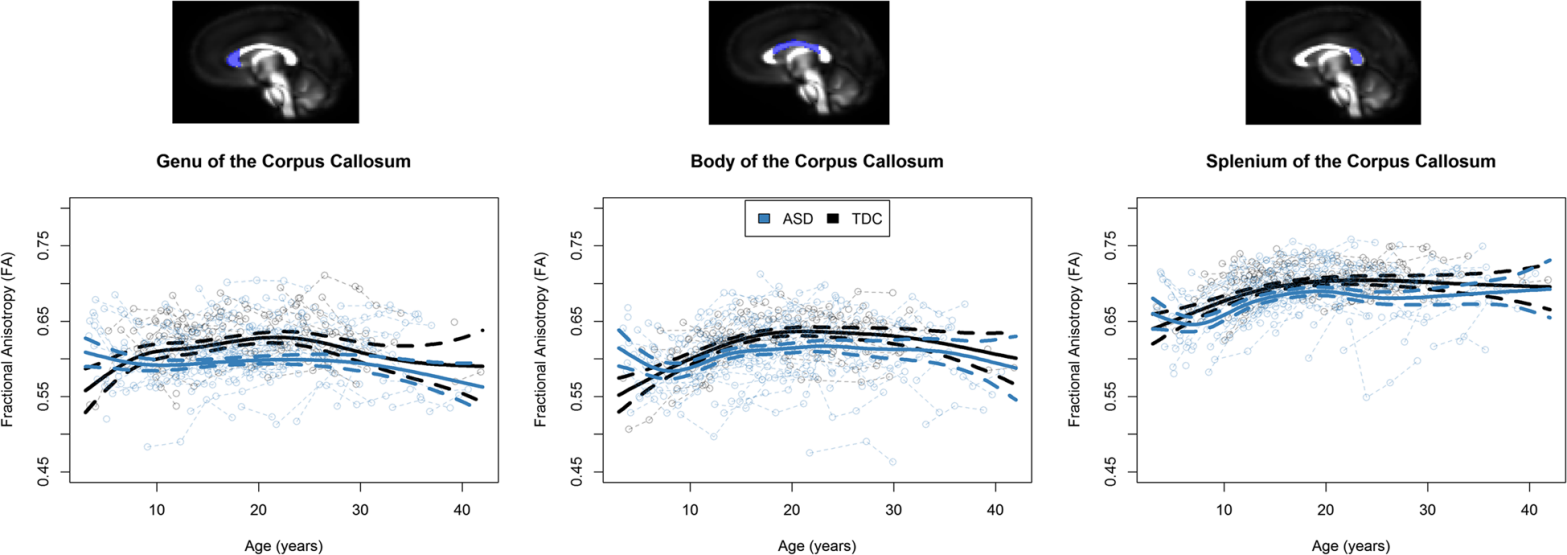
**Fractional anisotropy (FA) measures plotted as a function of age and group.** ASD, autism spectrum disorder; TDC, typically developing controls. Time 1 scatter has been adjusted for the head coil changes, and the overlaid growth models for each group represent the generalized additive mixed model spline curves (with dashed 95% confidence intervals), controlling for head coil changes, total motion index, signal-to-noise ratio, and random effects.

We tested for case-control differences in the tensor coefficients (FA, MD, RD, AD) and their longitudinal trajectories. FA measurements obtained from each of the three subregions of the corpus callosum (that is, the genu, body, and splenium) can be viewed in Figure 2. A figure of the cortical projections of each subregion of the corpus callosum can be seen in Additional file 2, which shows that the genu primarily contains projections to prefrontal cortices, the body contains projections premotor and supplementary motor frontal cortices to the primary somatosensory cortices, and the splenium contains projections to the parietal, occipital, and temporal lobes.

A generalized additive mixed-effects model analysis suggested decreased mean FA values in individuals with ASD in each of the corpus callosum subregions (see Table 2). These significant group differences in mean FA values were accompanied by a significant term for the age spline curve. In the genu and body, there were significant age-spline-by-group interactions, suggesting that the developmental trajectories of FA in those regions were different in the ASD group compared to the typically developing group. Follow-up linear mixed-effects models in different age groups (less than 10 years, 10 to 20 years, and over 20 years) revealed significant age-by-group interactions for FA in all subregions for individuals under 10 years of age, but there were no significant interactions for either of the older groups (Table 3).

**Table 3:** **Results of standard linear mixed-effects models for different age groups within the sample**

	**Intercept**	**Group**	***P*** **-value**	**Age**	***P*** **-value**	**Age X Group**	***P*** **-value**
Genu FA							
<10 years old	0.73	−0.22^a^	<0.001	0.010	0.03	−0.0189	0.002
10 to 20 years old	0.63	−0.02	0.04	0.001	0.48	0.0002	0.89
>20 years old	0.65	−0.04	0.004	−0.003	0.004	0.0019	0.09
Body FA							
<10 years old	0.69	−0.15	0.003	0.009	0.02	−0.0129	0.008
10 to 20 years old	0.64	−0.02	0.01	0.005	<0.001	−0.0019	0.12
>20 years old	0.65	−0.03	0.03	−0.002	0.03	0.0009	0.45
Splenium FA							
<10 years old	0.77	−0.15	0.006	0.008	0.06	−0.0110	0.03
10 to 20 years old	0.71	−0.01	0.11	0.002	0.01	0.0001	0.94
>20 years old	0.72	−0.02	0.01	−0.001	0.23	0.0010	0.26
Genu MD							
<10 years old	0.66	0.15	0.008	−0.011	0.02	0.0130	0.02
10 to 20 years old	0.75	0.01	0.22	−0.001	0.19	−0.0002	0.85
>20 years old	0.72	0.02	0.07	0.002	0.008	−0.0008	0.41
Body MD							
<10 years old	0.76	0.08	0.12	−0.006	0.14	0.0050	0.30
10 to 20 years old	0.78	0.01	0.24	−0.003	0.007	−0.0004	0.75
>20 years old	0.76	0.02	0.12	0.001	0.19	−0.0004	0.74
Splenium MD							
<10 years old	0.75	0.07	0.30	−0.003	0.57	0.0028	0.63
10 to 20 years old	0.77	0.01	0.40	−0.001	0.28	0.0004	0.73
>20 years old	0.74	0.02	0.048	0.002	0.01	−0.0011	0.28
Genu RD							
<10 years old	0.23	0.28	0.001	−0.014	0.04	0.0236	0.005
10 to 20 years old	0.37	0.02	0.10	−0.001	0.34	−0.0005	0.74
>20 years old	0.32	0.04	0.009	0.004	0.004	−0.0020	0.15
Body RD							
<10 years old	0.34	0.14	0.006	−0.010	0.02	0.0115	0.02
10 to 20 years old	0.38	0.02	0.03	−0.005	<0.001	0.0009	0.48
>20 years old	0.35	0.03	0.03	0.003	0.02	−0.0009	0.48
Splenium RD							
<10 years old	0.26	0.17	0.01	−0.006	0.22	0.0113	0.08
10 to 20 years old	0.30	0.01	0.10	−0.002	0.049	−0.0001	0.92
>20 years old	0.28	0.03	0.007	0.002	0.04	−0.0013	0.26
Genu AD							
<10 years old	1.35	−0.03	0.68	−0.008	0.12	−0.0019	0.75
10 to 20 years old	1.40	−0.01	0.20	−0.001	0.41	−0.0012	0.54
>20 years old	1.36	−0.01	0.66	0.000	0.76	0.0005	0.70
Body AD							
<10 years old	1.47	0.03	0.73	−0.004	0.54	−0.0012	0.87
10 to 20 years old	1.51	−0.03	0.02	0.003	0.15	−0.0054	0.02
>20 years old	1.49	−0.02	0.30	−0.002	0.15	0.0014	0.41
Splenium AD							
<10 years old	1.61	−0.07	0.30	0.007	0.18	−0.0091	0.17
10 to 20 years old	1.56	−0.01	0.33	0.001	0.51	−0.0003	0.86
>20 years old	1.53	0.01	0.56	0.001	0.71	−0.0003	0.87

Age groups were defined as less than 10 years old, 10 to 20 years old, and greater than 20 years old. The analyses in those under 10 years of age included 34 individuals with ASD (48 scans) and 12 individuals with typical development (17 scans). The analyses in those between 10 and 20 years of age included 61 individuals with ASD (127 scans) and 34 individuals with typical development (67 scans). The analyses in those over 20 years of age included 46 individuals with ASD (112 scans) and 29 individuals with typical development (63 scans). The mixed effects models examined diagnostic group main effects (ASD versus typical development), age main effects, and age-by-group interactions for FA, MD, RD, and AD of the genu, body, and splenium of the corpus callosum. The mixed effect model was denoted as DTI metric ~ 1 + group + age (years) + group × Age + (1 | participant), while controlling for head motion, SNR, and head coil. All intercepts were statistically significant. ^a^While this group effect is negative, caution should be employed when interpreting the direction of main effects in the presence of significant interactions. Graphically, the data demonstrate that most of the children under the age of 10 in the ASD group have higher levels of FA than in the typically developing group.

**Figure 3:**
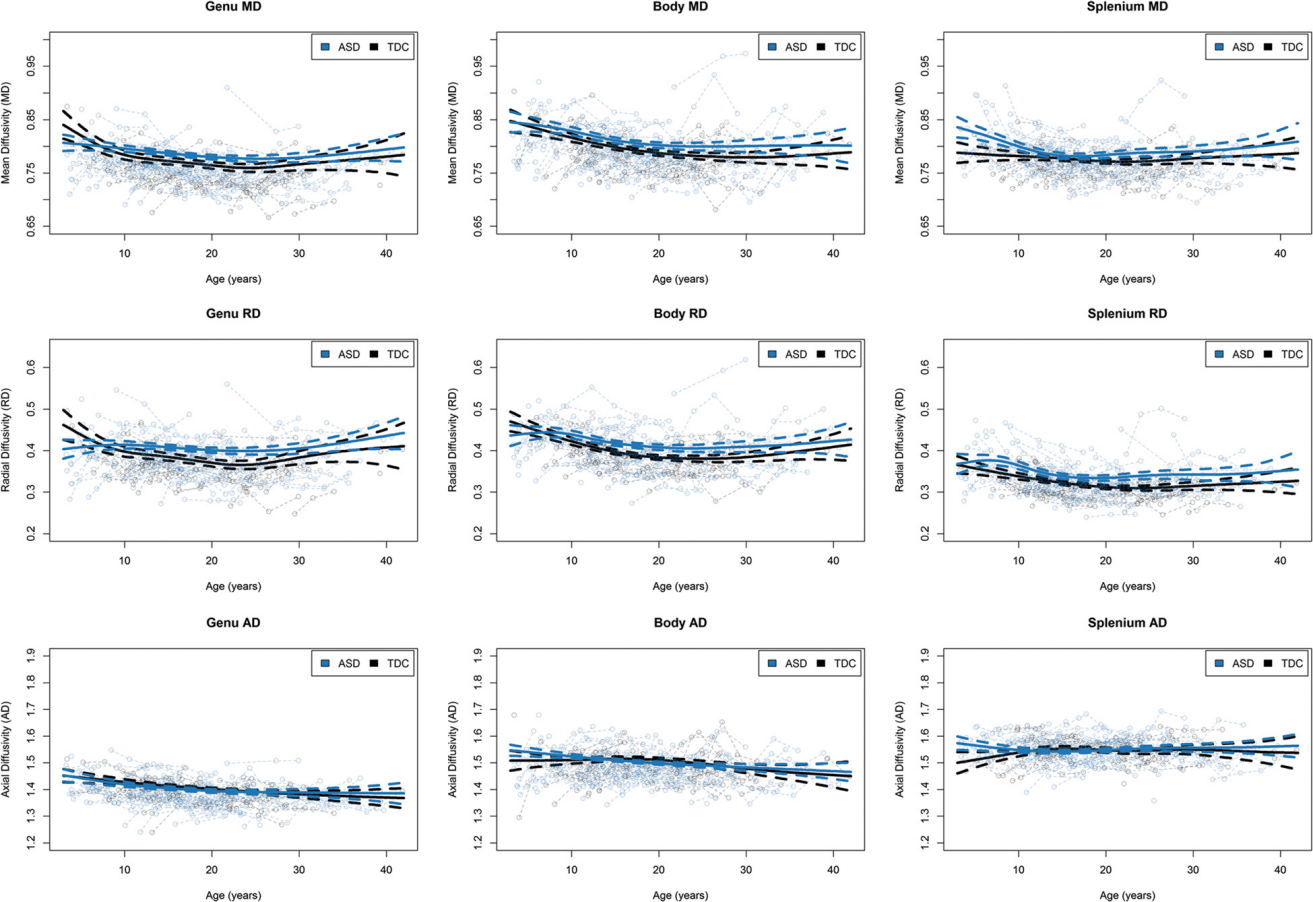
**Mean diffusivity (MD), radial diffusivity (RD), and axial diffusivity (AD) measures plotted as function of age and group.** ASD, autism spectrum disorder; TDC, typically developing controls. Time 1 scatter has been adjusted for the head coil changes, and the overlaid growth models for each group represent the generalized additive mixed model spline curves (with dashed 95% confidence intervals), controlling for head coil changes, total motion index, signal-to-noise ratio, and random effects.

Plots of MD, RD, and AD measurements obtained from the corpus callosum subregions are provided in Figure 3. Table 2 demonstrates significant group differences only in MD, RD, and AD of the splenium. However, it should be noted that even though splenium AD had a statistically significant group difference, it corresponded to a very small effect size (that is, on a 0.35% increase in AD in ASD), which means that it might not be a meaningful difference. The only significant age-by-group interaction was found in MD of the splenium. As in the FA follow-up results, linear mixed-effects models suggested that the majority of interaction effects occurred in the youngest age group (that is, participants less than 10 years of age).

### Differential analyses

Follow-up analyses examined these same models under four separate conditions: 1) time 1 only (using 8-channel head coil), 2) times 2 to 4 (using the 12-channel head coil), 3) without participants who were on centrally active medications, and 4) without participants who were sedated during scanning. The figures and tables of results for these differential analyses can be seen in the supplementary materials (Additional file 1). Without individuals who were medicated, the pattern of results was virtually identical. Similarly, when only time 1 was analyzed, similarly sized main effects and interactions were present (although some were only marginally significant due to the loss of three fourths of the data). However, without the individuals who were sedated during scanning (mostly younger participants) and without the data from time 1 (when most of the younger children’s data was collected), significant interaction effects were no longer present. Therefore, under conditions in which younger children with ASD were not as represented in the model, there were no differences in the developmental trajectories between the ASD group and the typically developing group. Nevertheless, because many of the younger children’s scans are confounded with sedation effects, the present study cannot definitively disentangle atypical early development of the corpus callosum from sedation effects in these models.

## Discussion

The present study used a cohort sequential design and generalized additive spline models to examine age-related trajectories of white matter microstructure of the corpus callosum in ASD compared to typical development. We aimed to longitudinally investigate development of corpus callosum microstructure in ASD from 3 years of age through the final stages of corpus callosum development in young adulthood. Even after controlling for known confounds in the imaging literature such as head motion and SNR, the longitudinal results yielded significant group differences in the microstructure of corpus callosum across the entire period. FA appeared to be atypically increased during early childhood. Its developmental trajectory during the first 10 years of life was in the opposite direction of typical development; FA decreased during childhood in the ASD group but increased as expected in TDC children. The ASD and TDC FA curves crossed during childhood, leading to a sustained decrease in FA in the ASD group relative to TDC during adolescence and young adulthood. Developmental abnormalities were found in all three subdivisions of the corpus callosum examined, predominantly affecting FA in the genu and body and MD in the splenium. These overall observations are bolstered by relative individual consistency of the longitudinal DTI measurements across the study and the consistency of these results with other investigations.

### Developmental differences in microstructure of corpus callosum

The present study demonstrated significant group differences in the developmental trajectory of the microstructure of the genu and body (FA). This atypical developmental trajectory in ASD was characterized by atypically high FA values in early childhood that decreased with age, crossed the TDC curve, and plateaued below the FA values of the typically developing group. Follow-up analyses conducted within particular age brackets reiterated that the developmental slope of FA was atypical only in the youngest ASD cohort (less than 10 years of age). This result is consistent with the only previous longitudinal investigation of corpus callosum microstructure in ASD [44], which examined microstructure in infants (6 to 24 months of age) and found atypical developmental trajectories of FA in the corpus callosum of the group that went on to develop ASD. Our sample did not overlap in age with the Wolff *et al*. study [44], but our results converge to suggest higher corpus callosum FA in early childhood in ASD that may decrease with age. Cross-sectional studies have also found increased FA in young children with autism [32,35,75,76]. Our result of crossing of the ASD and TDC corpus callosum FA growth curves at about 7 years of age is consistent with age-related findings in a cross-sectional study that suggested crossing between 7 and 8 years of age [76]. Intersecting of the corpus callosum FA growth curve during late childhood is consistent with cross-sectional studies at this age that have found no group differences [77,78].

We also found atypical longitudinal development of corpus callosum microstructure during adolescence and young adulthood. FA of the genu and body was persistently decreased; rate of change was similar to the TDC group. Cross-sectional studies of ASD have consistently found decreased FA in adolescents and adults with ASD [27-31,79-85]. The longitudinal trajectories depicted in our models of corpus callosum FA demonstrate that the inconsistencies that have been noted in the corpus callosum DTI in ASD literature are likely due to the different age ranges of the different studies. The present group differences are also consistent with functional connectivity findings of decreased interhemispheric connectivity in ASD [2,17-19]. It is possible that the present findings of decreased structural connectivity of the corpus callosum in ASD may correspond to these functional differences, although a direct comparison will be needed to confirm this relation. Recent evidence suggests that the relationship between structural connectivity indexed by DTI and resting state functional connectivity across the lifespan is complex [86].

In addition to differences in FA, the only corpus callosum region that the ASD group exhibited significantly increased MD, RD, and AD was in the splenium, although the effect size of the splenium AD was much smaller than the other effects. Further, there was a significant group difference in the developmental trajectory of splenium MD, but this should be interpreted with caution because our follow-up analyses did not converge to show that this was a significant effect in any of our age brackets. Although DTI measures do not specify the type of white matter microstructural changes that may be present (for example, myelination, axonal size and packing, or glial cellularity), the present results (decreased FA the splenium in conjunction with increased MD and RD [but smaller effect sized for group differences in AD]) are consistent with abnormal myelination in ASD [24]. Speculatively, factors such as atypical early development or maintenance of myelin (occurring before birth or in the first few years of life) could be underlying these brain differences in ASD. These mechanisms may be better elucidated in future research through animal and human postmortem myelination studies or by combining *in vivo* DTI with more specific imaging measures of myelination, including multicomponent relaxometry and magnetization transfer [24]. Although MD and RD of the genu did not exhibit significant group differences, it should be noted that these non-significant effects were in the same direction as the splenium effects. Therefore, it may be reasonable to assume that the underlying mechanisms may be similar across the corpus callosum.

It is further important to note that we observed great variability in the corpus callosum DTI values of the ASD group. As can be seen in the individual data points presented in Figure 2, there were a number of individuals with ASD who had typical corpus callosum values and a subset of individuals with ASD who demonstrated atypically low FA, high MD, and high RD. In the time 1 analyses of this data, Alexander *et al*. noted that group differences in the corpus callosum DTI metrics appeared to be driven by a minority of ASD participants [27]. The present longitudinal results extend upon those findings to show that the heterogeneity in the ASD group remained and that this subset of participants continued to show atypical DTI values across the 9-year period of this study. Future studies will investigate behavioral and genetic contributions to the variation in FA/MD in the participants with extreme corpus callosum values.

### The role of the corpus callosum in autism

Corpus callosum group differences have been the most consistently reported finding in the DTI in ASD literature [25]. Additionally, the corpus callosum repeatedly has been shown to be smaller in ASD [15,16], and functional imaging studies have demonstrated correlations between functional connectivity measures and the size of relevant regions of the corpus callosum [2,87-89]. The present longitudinal results reiterate corpus callosum pathology in ASD, and while white matter outside of the corpus callosum has been found to be affected in ASD, the corpus callosum should be given special consideration because of its size and of its participation in more global brain development. Specifically, the corpus callosum is the largest white matter tract in the brain, and it is known to reciprocally affect cortical development (see [46] for a review), which means that the corpus callosum could be intimately involved in the development of other structural brain differences that are observed in ASD. Indeed, atypical development of cortical thickness has been longitudinally observed in this sample [90], and future studies are critically needed to examine if corpus callosum atypicalities precede or co-occur with cortical thickness atypicalities in ASD.

Although there is converging evidence across multiple lines of research that suggest that the corpus callosum is commonly affected in ASD, the critical question of how corpus callosum atypicalities relate to the core behavioral symptoms of ASD remains. The similarity in some of the cognitive and symptom profiles between individuals with callosal agenesis and individuals with ASD is intriguing [91-93]. However, neither the literature nor the present study has established a one-to-one link between core ASD symptomatology and the corpus callosum. Perhaps, this is driven by the diversity of cognitive functions related to the corpus callosum [6], the development of compensatory mechanisms to accommodate corpus callosum atypicalities [94], the heterogeneity of symptom profiles within the ASD diagnosis, or the way in which corpus callosum development may be affecting or affected by cortical development [46]. Future research is needed to investigate how variation in specific neurocognitive functions may relate to variation in corpus callosum microstructure. Longitudinal studies provide the opportunity to discern causal pathways between alterations of brain structure, microstructure, and function and cognitive and behavioral impairments. Elucidation of causal pathways and mediating mechanisms, and development of predictive models, will pave the way for the formation of new biologically informed interventions and their targeted use in specific ASD subgroups.

### Limitations

There are several potential limitations associated with this present study. First, a limitation of the cohort sequential design is the use of a multiple cohorts across ages, which may make this design less sensitive to age-specific changes during narrow windows of development. However, the cohort sequential design allowed us to more rapidly answer this key longitudinal question in autism than would have been possible with a longitudinal panel design, and the accelerated design also minimized potential cohort-related confounds [95]. A second possible limitation is that the DTI protocol was held as consistent as possible across all data collection in this longitudinal study, despite the fact that more optimal protocols (for example, more than 12 directions, isotropic spatial resolution) have been developed over the years. In addition, there were unforeseeable changes in the hardware (head coil) and subsequent product DTI pulse sequence (TE) between time 1 and time 2 that resulted in a systematic bias in DTI measures between these two time points. As a result, a covariate for scanner was included in the age-trajectory models, which appears to have removed the bias associated with the imaging time point. The corrected measures were highly consistent across all time points, and the models were very similar without individuals with medication and when only time 1 data were included. However, the interaction effects were not sustained when eliminating participants under sedation and only times 2 through 4 data (both strategies that eliminated our youngest participants), which means that we cannot definitively disambiguate between the effects of age or sedation on our group differences in developmental trajectories. Sedation during pediatric neuroimaging has been shown to be safe and effective [96], and while there is evidence that there could be minimal effects of sedation on DTI metrics in temporal white matter [97], it is unclear whether this is due to the actual sedation or to the fact that the most severely impacted individuals with ASD were most likely to be sedated. Therefore, the effects of sedation on DTI are still unclear and will need to be elucidated in future research. Our study focused on verbal males to decrease heterogeneity in the ASD sample and increase statistical power. Additional studies of females and intellectually low-functioning individuals are needed to determine if the findings of the present study generalize to these groups. Finally, given the heterogeneity present even in verbal males with autism, replication of our findings in an independent sample is needed.

## Conclusions

The present study used DTI methods to longitudinally examine the white matter microstructure of the corpus callosum in individuals with ASD compared to typically developing controls (ages 3 to 41 years). The results suggest that corpus callosum microstructure (especially in the anterior corpus callosum) develops atypically in autism before the age of 10 years and that this early atypical development becomes a persistent group difference in later childhood, adolescence, and adulthood. Because the corpus callosum is the largest white matter tract in the brain, corpus callosum atypicalities likely have lasting effects on many aspects of cognition and brain synchronization in ASD.

## Additional files

Additional file 1: **Supplementary materials for differential analyses.** This file contains five tables (Tables S1-S5) and eight figures (Figures S1-S8) that present the results of our differential analyses.

Additional file 2: **Projections of the genu, body, and splenium subsections of the corpus callosum.** This picture file demonstrates the cortical projections of the genu (red), body (green), and splenium (blue) subregions of the corpus callosum in our study’s template space.

## Abbreviations

AD: axial diffusivity
ADOS: Autism Diagnostic Observation Scale
ASD: autism Spectrum Disorder
DTI: diffusion tensor imaging
DWI: diffusion-weighted images
EPI: echo planar imaging
FA: fractional anisotropy
FOV: field of view
FSIQ: full-scale IQ
FWHM: full width at half maximum
ICC: intraclass correlation coefficient
MD: mean diffusivity
PIQ: performance IQ
RD: radial diffusivity
RESTORE: robust estimation algorithm
ROI: region of interest
SNR: signal-to-noise ratio
TE: echo time
TMI: total motion index
TR: repetition time
VIQ: verbal IQ
WASI: Wechsler Abbreviated Scale of Intelligence
