# Supplementary material for: Atypical development of white matter microstructure of the corpus callosum in males with autism: a longitudinal investigation
Source: Mol Autism. 2015 Mar 11;6:15. doi: 10.1186/s13229-015-0001-8 (PMC4359536; doi:10.1186/s13229-015-0001-8)
Supplement: Supplementary file 1 — Supplementary materials for differential analyses. This file contains five tables (Tables S1-S5) and eight figures (Figures S1-S8) that present the results of our differential analyses. [file 13229_2015_1_MOESM1_ESM.docx]

**Supplementary Materials**

**Table S1.** Time 1 (8 channel head coil) generalized additive model results. For the parametric group differences, the estimate is reported as the percent deviation in the ASD group compared to the TDC group with the corresponding p-value. For the age spline and the age-by-group splines, the estimated degrees of freedom (EDF), F-value, and p-values are reported.

|  |  | **Group** | | **Age Spline** | | | **Age X Group Spline** | | |
| --- | --- | --- | --- | --- | --- | --- | --- | --- | --- |
|  |  | **Estimate** | **p-value** | **EDF** | **F** | **p-value** | **EDF** | **F** | **p-value** |
| **Genu FA** |  |  |  |  |  |  |  |  |  |
|  | ASD & TDC | -3.91% | 0.07 | 1.00 | 6.83 | 0.01 | 3.86 | 2.77 | 0.02 |
|  | ASD | -- | -- | 3.44 | 0.99 | 0.42 | -- | -- | -- |
|  | TDC | -- | -- | 1.00 | 13.01 | <.001 | -- | -- | -- |
| **Body FA** |  |  |  |  |  |  |  |  |  |
|  | ASD & TDC | -3.69% | 0.24 | 1.33 | 3.77 | 0.04 | 5.50 | 1.63 | 0.14 |
|  | ASD | -- | -- | 1.80 | 0.64 | 0.55 | -- | -- | -- |
|  | TDC | -- | -- | 2.09 | 6.84 | 0.001 | -- | -- | -- |
| **Splenium FA** |  |  |  |  |  |  |  |  |  |
|  | ASD & TDC | -4.10% | 0.17 | 1.00 | 12.01 | <.001 | 6.09 | 2.85 | 0.01 |
|  | ASD | -- | -- | 5.72 | 2.45 | 0.02 | -- | -- | -- |
|  | TDC | -- | -- | 1.00 | 17.12 | <.001 | -- | -- | -- |
| **Genu MD** |  |  |  |  |  |  |  |  |  |
|  | ASD & TDC | 2.85% | 0.24 | 4.61 | 4.10 | 0.001 | 1.00 | 5.04 | 0.03 |
|  | ASD | -- | -- | 1.86 | 2.39 | 0.09 | -- | -- | -- |
|  | TDC | -- | -- | 2.80 | 13.66 | <.001 | -- | -- | -- |
| **Body MD** |  |  |  |  |  |  |  |  |  |
|  | ASD & TDC | 3.59% | 0.81 | 1.00 | 2.89 | 0.09 | 4.94 | 2.10 | 0.06 |
|  | ASD | -- | -- | 2.31 | 2.93 | 0.04 | -- | -- | -- |
|  | TDC | -- | -- | 1.00 | 12.76 | <.001 | -- | -- | -- |
| **Splenium MD** |  |  |  |  |  |  |  |  |  |
|  | ASD & TDC | 3.46% | 0.48 | 1.00 | 2.99 | 0.09 | 6.22 | 2.65 | 0.01 |
|  | ASD | -- | -- | 5.66 | 4.36 | <.001 | -- | -- | -- |
|  | TDC | -- | -- | 1.00 | 9.99 | 0.003 | -- | -- | -- |
| **Genu RD** |  |  |  |  |  |  |  |  |  |
|  | ASD & TDC | 8.14% | 0.06 | 1.00 | 11.55 | <.001 | 3.89 | 2.92 | 0.02 |
|  | ASD | -- | -- | 3.26 | 0.84 | 0.51 | -- | -- | -- |
|  | TDC | -- | -- | 2.49 | 9.23 | <.001 | -- | -- | -- |
| **Body RD** |  |  |  |  |  |  |  |  |  |
|  | ASD & TDC | 8.16% | 0.80 | 5.47 | 2.68 | 0.01 | 1.00 | 1.43 | 0.23 |
|  | ASD | -- | -- | 4.69 | 1.68 | 0.14 | -- | -- | -- |
|  | TDC | -- | -- | 1.77 | 8.28 | <.001 | -- | -- | -- |
| **Splenium RD** |  |  |  |  |  |  |  |  |  |
|  | ASD & TDC | 12.11% | 0.26 | 1.00 | 6.58 | 0.01 | 5.79 | 2.45 | 0.02 |
|  | ASD | -- | -- | 5.31 | 2.22 | 0.04 | -- | -- | -- |
|  | TDC | -- | -- | 1.00 | 16.18 | <.001 | -- | -- | -- |
| **Genu AD** |  |  |  |  |  |  |  |  |  |
|  | ASD & TDC | 0.55% | 0.55 | 2.04 | 4.17 | 0.01 | 1.00 | 0.31 | 0.58 |
|  | ASD | -- | -- | 1.77 | 6.94 | <.001 | -- | -- | -- |
|  | TDC | -- | -- | 1.56 | 4.35 | 0.02 | -- | -- | -- |
| **Body AD** |  |  |  |  |  |  |  |  |  |
|  | ASD & TDC | 0.89% | 0.03 | 6.95 | 1.12 | 0.35 | 2.39 | 2.58 | 0.06 |
|  | ASD | -- | -- | 1.88 | 2.77 | 0.06 | -- | -- | -- |
|  | TDC | -- | -- | 1.84 | 1.12 | 0.34 | -- | -- | -- |
| **Splenium AD** |  |  |  |  |  |  |  |  |  |
|  | ASD & TDC | 0.09% | 0.04 | 1.00 | 0.96 | 0.33 | 2.06 | 2.12 | 0.11 |
|  | ASD | -- | -- | 1.72 | 3.32 | 0.04 | -- | -- | -- |
|  | TDC | -- | -- | 2.17 | 0.78 | 0.49 | -- | -- | -- |

AD= Axial diffusivity,; ASD= Autism Spectrum Disorder, EDF = Estimated degrees of freedom to assess smooth spline complexity; ICC= intra-class correlation coefficient; FA= Fractional anisotropy; MD = Mean diffusivity; RD = Radial Diffusivity; TDC = Typically developing controls.

**Table S2.** Times 2 through 4 (12 channel head coil) generalized additive mixed model results. For the parametric group differences, the estimate is reported as the percent deviation in the ASD group compared to the TDC group with the corresponding p-value. For the age spline and the age-by-group splines, the estimated degrees of freedom (EDF), F-value, and p-values are reported.

|  |  | **Group** | | **Age Spline** | | | | **Age X Group Spline** | | | |  |
| --- | --- | --- | --- | --- | --- | --- | --- | --- | --- | --- | --- | --- |
|  |  | **Estimate** | **p-value** | | **EDF** | **F** | **p-value** | | **EDF** | **F** | **p-value** | |
| **Genu FA** |  |  |  | |  |  |  | |  |  |  | |
|  | ASD & TDC | -2.74% | 0.33 | | 3.91 | 5.05 | <.001 | | 1.00 | 0.10 | 0.75 | |
|  | ASD | -- | -- | | 3.28 | 3.98 | 0.01 | | -- | -- | -- | |
|  | TDC | -- | -- | | 5.21 | 2.90 | 0.02 | | -- | -- | -- | |
| **Body FA** |  |  |  | |  |  |  | |  |  |  | |
|  | ASD & TDC | -1.81% | 0.69 | | 5.87 | 14.48 | <.001 | | 1.00 | 0.60 | 0.44 | |
|  | ASD | -- | -- | | 4.64 | 8.22 | <.001 | | -- | -- | -- | |
|  | TDC | -- | -- | | 4.48 | 14.51 | <.001 | | -- | -- | -- | |
| **Splenium FA** |  |  |  | |  |  |  | |  |  |  | |
|  | ASD & TDC | -1.38% | 0.13 | | 5.06 | 12.88 | <.001 | | 1.00 | 0.12 | 0.73 | |
|  | ASD | -- | -- | | 4.57 | 10.01 | <.001 | | -- | -- | -- | |
|  | TDC | -- | -- | | 4.36 | 9.82 | <.001 | | -- | -- | -- | |
| **Genu MD** |  |  |  | |  |  |  | |  |  |  | |
|  | ASD & TDC | 1.14% | 0.58 | | 4.89 | 5.86 | <.001 | | 1.20 | 0.11 | 0.79 | |
|  | ASD | -- | -- | | 3.97 | 7.08 | <.001 | | -- | -- | -- | |
|  | TDC | -- | -- | | 4.24 | 1.83 | 0.12 | | -- | -- | -- | |
| **Body MD** |  |  |  | |  |  |  | |  |  |  | |
|  | ASD & TDC | 1.30% | 0.66 | | 4.56 | 15.38 | <.001 | | 1.00 | 0.21 | 0.65 | |
|  | ASD | -- | -- | | 4.20 | 10.76 | <.001 | | -- | -- | -- | |
|  | TDC | -- | -- | | 3.62 | 14.75 | <.001 | | -- | -- | -- | |
| **Splenium MD** |  |  |  | |  |  |  | |  |  |  | |
|  | ASD & TDC | 1.25% | 0.47 | | 3.12 | 2.78 | 0.04 | | 1.39 | 0.17 | 0.76 | |
|  | ASD | -- | -- | | 3.19 | 3.32 | 0.02 | | -- | -- | -- | |
|  | TDC | -- | -- | | 2.39 | 1.18 | 0.31 | | -- | -- | -- | |
| **Genu RD** |  |  |  | |  |  |  | |  |  |  | |
|  | ASD & TDC | 4.89% | 0.56 | | 5.31 | 6.25 | <.001 | | 1.00 | 0.35 | 0.55 | |
|  | ASD | -- | -- | | 3.94 | 5.75 | <.001 | | -- | -- | -- | |
|  | TDC | -- | -- | | 5.62 | 2.71 | 0.02 | | -- | -- | -- | |
| **Body RD** |  |  |  | |  |  |  | |  |  |  | |
|  | ASD & TDC | 4.24% | 0.38 | | 5.47 | 17.47 | <.001 | | 1.00 | 0.12 | 0.72 | |
|  | ASD | -- | -- | | 4.59 | 10.34 | <.001 | | -- | -- | -- | |
|  | TDC | -- | -- | | 4.53 | 17.67 | <.001 | | -- | -- | -- | |
| **Splenium RD** |  |  |  | |  |  |  | |  |  |  | |
|  | ASD & TDC | 5.40% | 0.09 | | 4.29 | 5.06 | <.001 | | 2.12 | 0.73 | 0.49 | |
|  | ASD | -- | -- | | 4.36 | 7.26 | <.001 | | -- | -- | -- | |
|  | TDC | -- | -- | | 3.86 | 5.51 | <.001 | | -- | -- | -- | |
| **Genu AD** |  |  |  | |  |  |  | |  |  |  | |
|  | ASD & TDC | -0.63% | 0.21 | | 2.79 | 6.02 | <.001 | | 1.00 | 0.84 | 0.36 | |
|  | ASD | -- | -- | | 2.45 | 3.55 | 0.02 | | -- | -- | -- | |
|  | TDC | -- | -- | | 2.00 | 7.94 | <.001 | | -- | -- | -- | |
| **Body AD** |  |  |  | |  |  |  | |  |  |  | |
|  | ASD & TDC | 0.26% | 0.22 | | 1.00 | 9.86 | 0.002 | | 2.83 | 1.61 | 0.19 | |
|  | ASD | -- | -- | | 2.78 | 7.67 | <.001 | | -- | -- | -- | |
|  | TDC | -- | -- | | 1.00 | 10.46 | 0.002 | | -- | -- | -- | |
| **Splenium AD** |  |  |  | |  |  |  | |  |  |  | |
|  | ASD & TDC | 0.50% | 0.98 | | 1.00 | 0.22 | 0.64 | | 1.00 | 0.13 | 0.72 | |
|  | ASD | -- | -- | | 1.00 | 1.21 | 0.27 | | -- | -- | -- | |
|  | TDC | -- | -- | | 3.07 | 1.34 | 0.27 | | -- | -- | -- | |

AD= Axial diffusivity; ASD= Autism Spectrum Disorder, EDF = Estimated degrees of freedom to assess smooth spline complexity; ICC= intra-class correlation coefficient; FA= Fractional anisotropy; MD = Mean diffusivity; RD = Radial Diffusivity; TDC = Typically developing controls.

**Table S3.** Generalized additive mixed model results for all time points, excluding scans where participants with ASD were on centrally active medications. For the parametric group differences, the estimate is reported as the percent deviation in the ASD group compared to the TDC group with the corresponding p-value. For the age spline and the age-by-group splines, the estimated degrees of freedom (EDF), F-value, and p-values are reported.

|  |  | **Group** | | **Age Spline** | | | **Group x Age Spline** | | |
| --- | --- | --- | --- | --- | --- | --- | --- | --- | --- |
|  |  | **Estimate** | **p-value** | **EDF** | **F** | **p-value** | **EDF** | **F** | **p-value** |
| **Genu FA** |  |  |  |  |  |  |  |  |  |
|  | ASD & TDC | -3.37% | 0.03 | 3.35 | 4.00 | 0.01 | 4.85 | 2.52 | 0.03 |
|  | ASD | -- | -- | 4.33 | 1.92 | 0.10 | -- | -- | -- |
|  | TDC | -- | -- | 5.26 | 5.02 | <.001 | -- | -- | -- |
| **Body FA** |  |  |  |  |  |  |  |  |  |
|  | ASD & TDC | -2.93% | 0.003 | 3.93 | 9.44 | <.001 | 6.03 | 2.14 | 0.05 |
|  | ASD | -- | -- | 5.54 | 3.12 | 0.01 | -- | -- | -- |
|  | TDC | -- | -- | 4.25 | 15.64 | <.001 | -- | -- | -- |
| **Splenium FA** |  |  |  |  |  |  |  |  |  |
|  | ASD & TDC | -2.57% | 0.01 | 6.32 | 7.58 | <.001 | 1.00 | 1.04 | 0.31 |
|  | ASD | -- | -- | 7.08 | 7.55 | <.001 | -- | -- | -- |
|  | TDC | -- | -- | 3.67 | 11.54 | <.001 | -- | -- | -- |
| **Genu MD** |  |  |  |  |  |  |  |  |  |
|  | ASD & TDC | 2.21% | 0.26 | 3.34 | 7.60 | <.001 | 1.00 | 0.00 | 0.99 |
|  | ASD | -- | -- | 2.70 | 5.19 | 0.003 | -- | -- | -- |
|  | TDC | -- | -- | 4.87 | 7.06 | <.001 | -- | -- | -- |
| **Body MD** |  |  |  |  |  |  |  |  |  |
|  | ASD & TDC | 2.61% | 0.25 | 2.92 | 6.35 | <.001 | 2.27 | 0.34 | 0.74 |
|  | ASD | -- | -- | 3.33 | 6.85 | <.001 | -- | -- | -- |
|  | TDC | -- | -- | 3.06 | 12.13 | <.001 | -- | -- | -- |
| **Splenium MD** |  |  |  |  |  |  |  |  |  |
|  | ASD & TDC | 2.33% | 0.001 | 1.89 | 0.85 | 0.41 | 3.90 | 3.15 | 0.02 |
|  | ASD | -- | -- | 3.60 | 5.68 | <.001 | -- | -- | -- |
|  | TDC | -- | -- | 2.96 | 1.63 | 0.18 | -- | -- | -- |
| **Genu RD** |  |  |  |  |  |  |  |  |  |
|  | ASD & TDC | 7.09% | 0.13 | 3.31 | 4.05 | 0.01 | 4.15 | 1.65 | 0.16 |
|  | ASD | -- | -- | 3.77 | 1.51 | 0.20 | -- | -- | -- |
|  | TDC | -- | -- | 4.87 | 5.89 | <.001 | -- | -- | -- |
| **Body RD** |  |  |  |  |  |  |  |  |  |
|  | ASD & TDC | 6.86% | 0.69 | 4.45 | 10.83 | <.001 | 1.00 | 1.07 | 0.30 |
|  | ASD | -- | -- | 3.96 | 4.98 | <.001 | -- | -- | -- |
|  | TDC | -- | -- | 4.00 | 15.00 | <.001 | -- | -- | -- |
| **Splenium RD** |  |  |  |  |  |  |  |  |  |
|  | ASD & TDC | 8.74% | 0.002 | 4.38 | 6.21 | <.001 | 1.00 | 2.20 | 0.14 |
|  | ASD | -- | -- | 6.10 | 4.11 | <.001 | -- | -- | -- |
|  | TDC | -- | -- | 3.34 | 8.66 | <.001 | -- | -- | -- |
| **Genu AD** |  |  |  |  |  |  |  |  |  |
|  | ASD & TDC | 0.14% | 0.35 | 2.25 | 6.30 | 0.001 | 2.17 | 0.80 | 0.46 |
|  | ASD | -- | -- | 2.71 | 7.07 | <.001 | -- | -- | -- |
|  | TDC | -- | -- | 1.87 | 8.58 | <.001 | -- | -- | -- |
| **Body AD** |  |  |  |  |  |  |  |  |  |
|  | ASD & TDC | 0.32% | 0.10 | 1.00 | 5.19 | 0.02 | 2.01 | 1.66 | 0.19 |
|  | ASD | -- | -- | 1.80 | 10.88 | <.001 | -- | -- | -- |
|  | TDC | -- | -- | 2.66 | 3.25 | 0.03 | -- | -- | -- |
| **Splenium AD** |  |  |  |  |  |  |  |  |  |
|  | ASD & TDC | 0.48% | 0.18 | 1.00 | 0.51 | 0.48 | 1.80 | 1.16 | 0.30 |
|  | ASD | -- | -- | 1.77 | 0.56 | 0.55 | -- | -- | -- |
|  | TDC | -- | -- | 3.70 | 1.64 | 0.17 | -- | -- | -- |

AD= Axial diffusivity; ASD= Autism Spectrum Disorder, EDF = Estimated degrees of freedom to assess smooth spline complexity; ICC= intra-class correlation coefficient; FA= Fractional anisotropy; MD = Mean diffusivity; RD = Radial Diffusivity; TDC = Typically developing controls.

**Table S4.** Generalized additive mixed model results for all time points, excluding scans where the participants with ASD were sedated. For the parametric group differences, the estimate is reported as the percent deviation in the ASD group compared to the TDC group with the corresponding p-value. For the age spline and the age-by-group splines, the estimated degrees of freedom (EDF), F-value, and p-values are reported.

|  |  | **Group** | | **Age Spline** | | | **Group x Age Spline** | | |
| --- | --- | --- | --- | --- | --- | --- | --- | --- | --- |
|  |  | **Estimate** | **p-value** | **EDF** | **F** | **p-value** | **EDF** | **F** | **p-value** |
| **Genu FA** |  |  |  |  |  |  |  |  |  |
|  | ASD & TDC | -3.00% | 0.02 | 3.93 | 6.49 | <.001 | 1.00 | 0.96 | 0.33 |
|  | ASD | -- | -- | 2.78 | 2.61 | 0.06 | -- | -- | -- |
|  | TDC | -- | -- | 5.26 | 5.02 | <.001 | -- | -- | -- |
| **Body FA** |  |  |  |  |  |  |  |  |  |
|  | ASD & TDC | -1.81% | 0.39 | 4.11 | 9.74 | <.001 | 1.00 | 0.20 | 0.65 |
|  | ASD | -- | -- | 2.55 | 2.37 | 0.08 | -- | -- | -- |
|  | TDC | -- | -- | 4.25 | 15.64 | <.001 | -- | -- | -- |
| **Splenium FA** |  |  |  |  |  |  |  |  |  |
|  | ASD & TDC | -1.48% | 0.02 | 4.84 | 10.62 | <.001 | 1.00 | 0.81 | 0.37 |
|  | ASD | -- | -- | 4.44 | 5.41 | <.001 | -- | -- | -- |
|  | TDC | -- | -- | 3.67 | 11.54 | <.001 | -- | -- | -- |
| **Genu MD** |  |  |  |  |  |  |  |  |  |
|  | ASD & TDC | 1.16% | 0.30 | 5.16 | 6.66 | <.001 | 1.00 | 0.01 | 0.93 |
|  | ASD | -- | -- | 2.81 | 2.63 | 0.05 | -- | -- | -- |
|  | TDC | -- | -- | 4.87 | 7.06 | <.001 | -- | -- | -- |
| **Body MD** |  |  |  |  |  |  |  |  |  |
|  | ASD & TDC | 1.46% | 0.53 | 2.92 | 5.25 | 0.002 | 1.00 | 0.10 | 0.75 |
|  | ASD | -- | -- | 2.16 | 1.77 | 0.17 | -- | -- | -- |
|  | TDC | -- | -- | 3.06 | 12.13 | <.001 | -- | -- | -- |
| **Splenium MD** |  |  |  |  |  |  |  |  |  |
|  | ASD & TDC | 1.28% | 0.03 | 1.82 | 0.62 | 0.52 | 3.82 | 1.14 | 0.33 |
|  | ASD | -- | -- | 3.74 | 1.71 | 0.15 | -- | -- | -- |
|  | TDC | -- | -- | 2.96 | 1.63 | 0.18 | -- | -- | -- |
| **Genu RD** |  |  |  |  |  |  |  |  |  |
|  | ASD & TDC | 5.29% | 0.06 | 4.21 | 7.01 | <.001 | 1.00 | 0.36 | 0.55 |
|  | ASD | -- | -- | 2.95 | 3.26 | 0.02 | -- | -- | -- |
|  | TDC | -- | -- | 4.87 | 5.89 | <.001 | -- | -- | -- |
| **Body RD** |  |  |  |  |  |  |  |  |  |
|  | ASD & TDC | 4.15% | 0.29 | 3.86 | 9.12 | <.001 | 1.00 | 0.12 | 0.73 |
|  | ASD | -- | -- | 2.60 | 2.65 | 0.06 | -- | -- | -- |
|  | TDC | -- | -- | 4.00 | 15.00 | <.001 | -- | -- | -- |
| **Splenium RD** |  |  |  |  |  |  |  |  |  |
|  | ASD & TDC | 5.61% | 0.02 | 3.47 | 4.52 | 0.003 | 2.78 | 0.86 | 0.45 |
|  | ASD | -- | -- | 3.90 | 3.44 | 0.01 | -- | -- | -- |
|  | TDC | -- | -- | 3.34 | 8.66 | <.001 | -- | -- | -- |
| **Genu AD** |  |  |  |  |  |  |  |  |  |
|  | ASD & TDC | -0.79% | 0.21 | 1.67 | 5.41 | 0.01 | 1.00 | 0.84 | 0.36 |
|  | ASD | -- | -- | 1.00 | 3.63 | 0.06 | -- | -- | -- |
|  | TDC | -- | -- | 1.87 | 8.58 | <.001 | -- | -- | -- |
| **Body AD** |  |  |  |  |  |  |  |  |  |
|  | ASD & TDC | -0.09% | 0.77 | 1.52 | 3.28 | 0.05 | 1.00 | 0.03 | 0.87 |
|  | ASD | -- | -- | 1.00 | 4.40 | 0.04 | -- | -- | -- |
|  | TDC | -- | -- | 2.66 | 3.25 | 0.03 | -- | -- | -- |
| **Splenium AD** |  |  |  |  |  |  |  |  |  |
|  | ASD & TDC | 0.30% | 0.81 | 1.00 | 0.19 | 0.67 | 1.00 | 0.11 | 0.74 |
|  | ASD | -- | -- | 1.00 | 0.55 | 0.46 | -- | -- | -- |
|  | TDC | -- | -- | 3.70 | 1.64 | 0.17 | -- | -- | -- |

AD= Axial diffusivity; ASD= Autism Spectrum Disorder, EDF = Estimated degrees of freedom to assess smooth spline complexity; ICC= intra-class correlation coefficient; FA= Fractional anisotropy; MD = Mean diffusivity; RD = Radial Diffusivity; TDC = Typically developing controls.

**Table S5.** Number of participants who were on centrally active medications (Meds) and/or sedated at each of the Times.

|  | **Time 1 (8-channel head coil)** | |  |
| --- | --- | --- | --- |
|  | Sedated | Not Sedated | **Meds Totals** |
| Meds | 9 | 29 | **38** |
| No Meds | 23 | 74 | **97** |
| **Sedation Totals** | **32** | **103** |  |
|  |  |  |  |
|  | **Time 2 (12-channel head coil)** | |  |
|  | Sedated | Not Sedated | **Meds Totals** |
| Meds | 4 | 27 | **31** |
| No Meds | 11 | 68 | **79** |
| **Sedation Totals** | **15** | **95** |  |
|  |  |  |  |
|  | **Time 3 (12-channel head coil)** | |  |
|  | Sedated | Not Sedated | **Meds Totals** |
| Meds | 0 | 19 | **19** |
| No Meds | 0 | 88 | **88** |
| **Sedation Totals** | **0** | **107** |  |
|  |  |  |  |
|  | **Time 4 (12-channel head coil)** | |  |
|  | Sedated | Not Sedated | **Meds Totals** |
| Meds | 0 | 10 | **10** |
| No Meds | 0 | 73 | **73** |
| **Sedation Totals** | **0** | **83** |  |

**Figure S1**. Generalized additive model spline results for corpus callosum fractional anisotropy (FA) at Time 1 (8-channel head coil).

**
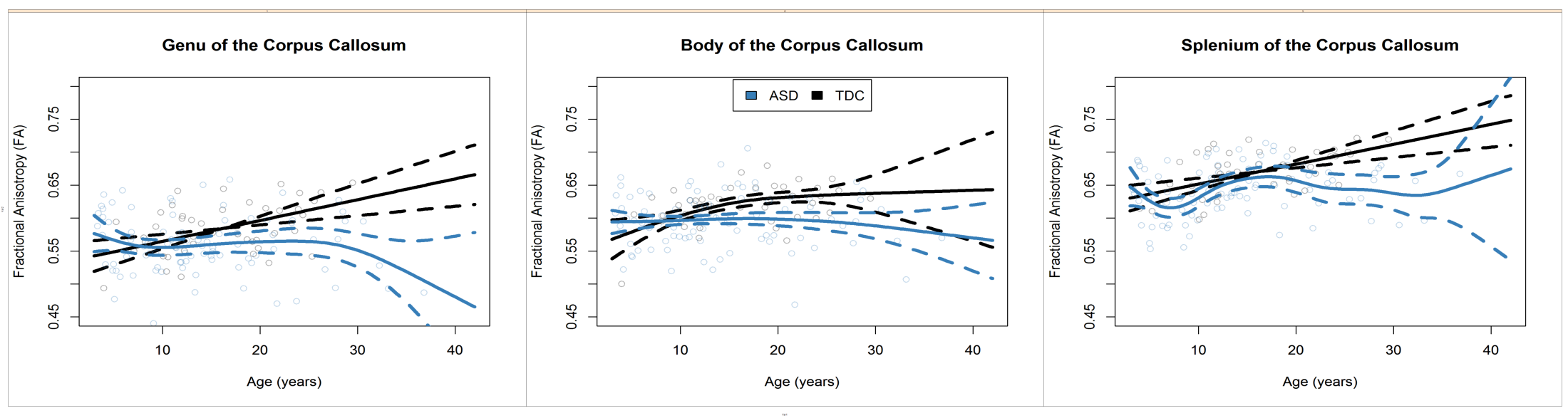
**


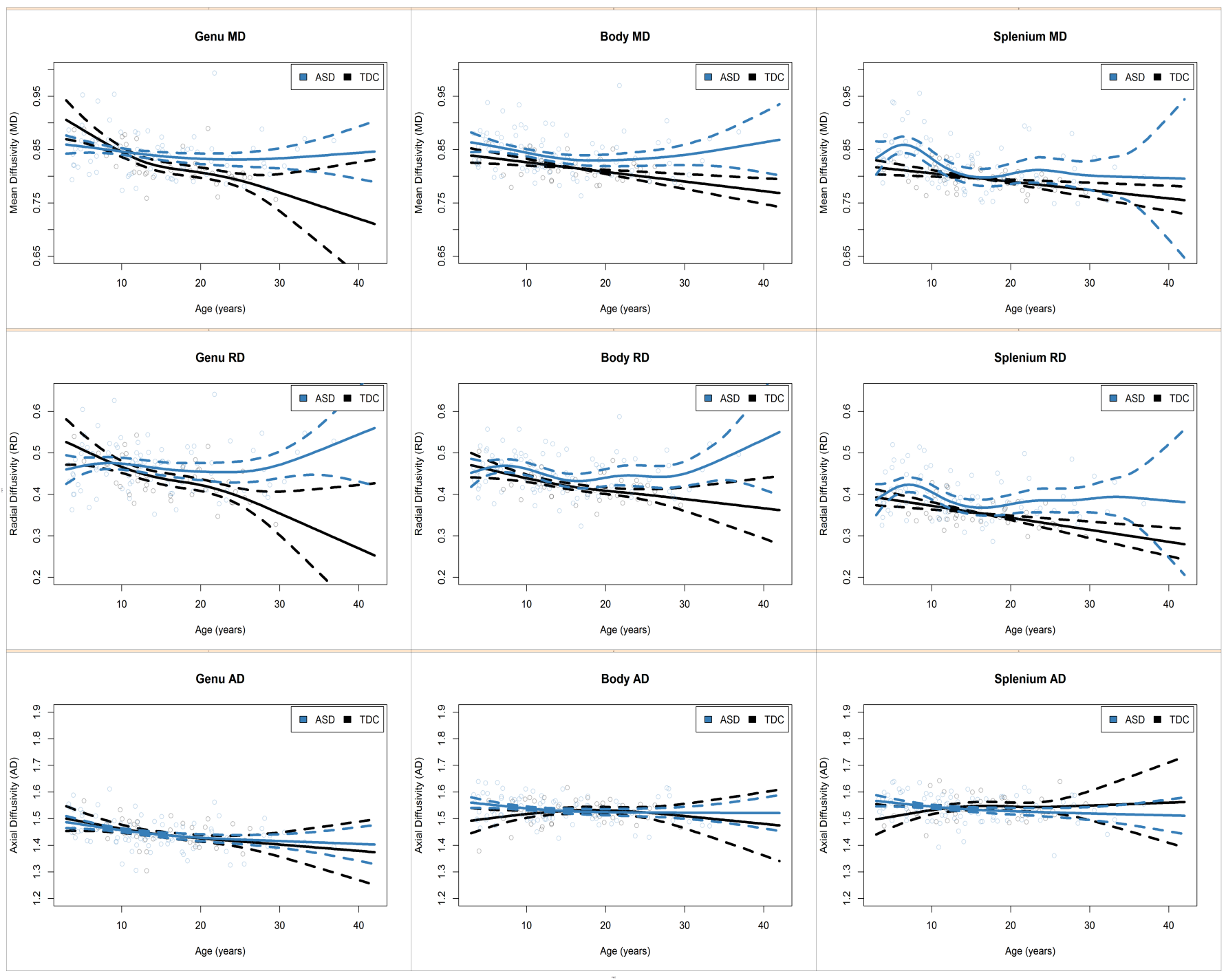
**Figure S2**. Generalized additive model spline results for corpus callosum mean diffusivity (MD), radial diffusivity (RD), and axial diffusivity (AD) at Time 1 (8-channel head coil).

**Figure S3**. Generalized additive mixed model spline results for corpus callosum fractional anisotropy (FA) for Times 2, 3, and 4 (12-channel head coil).


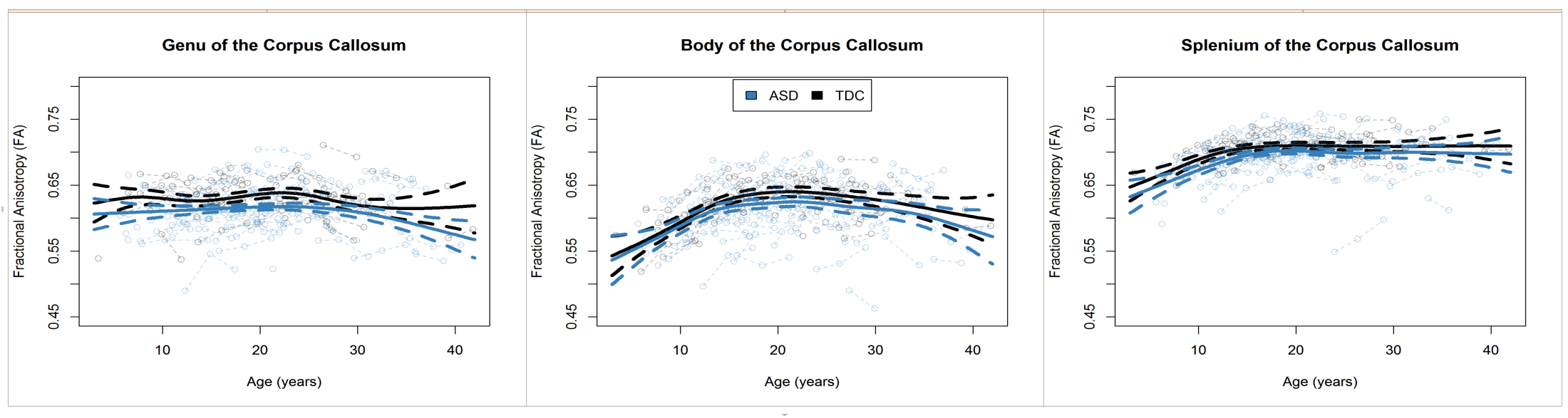


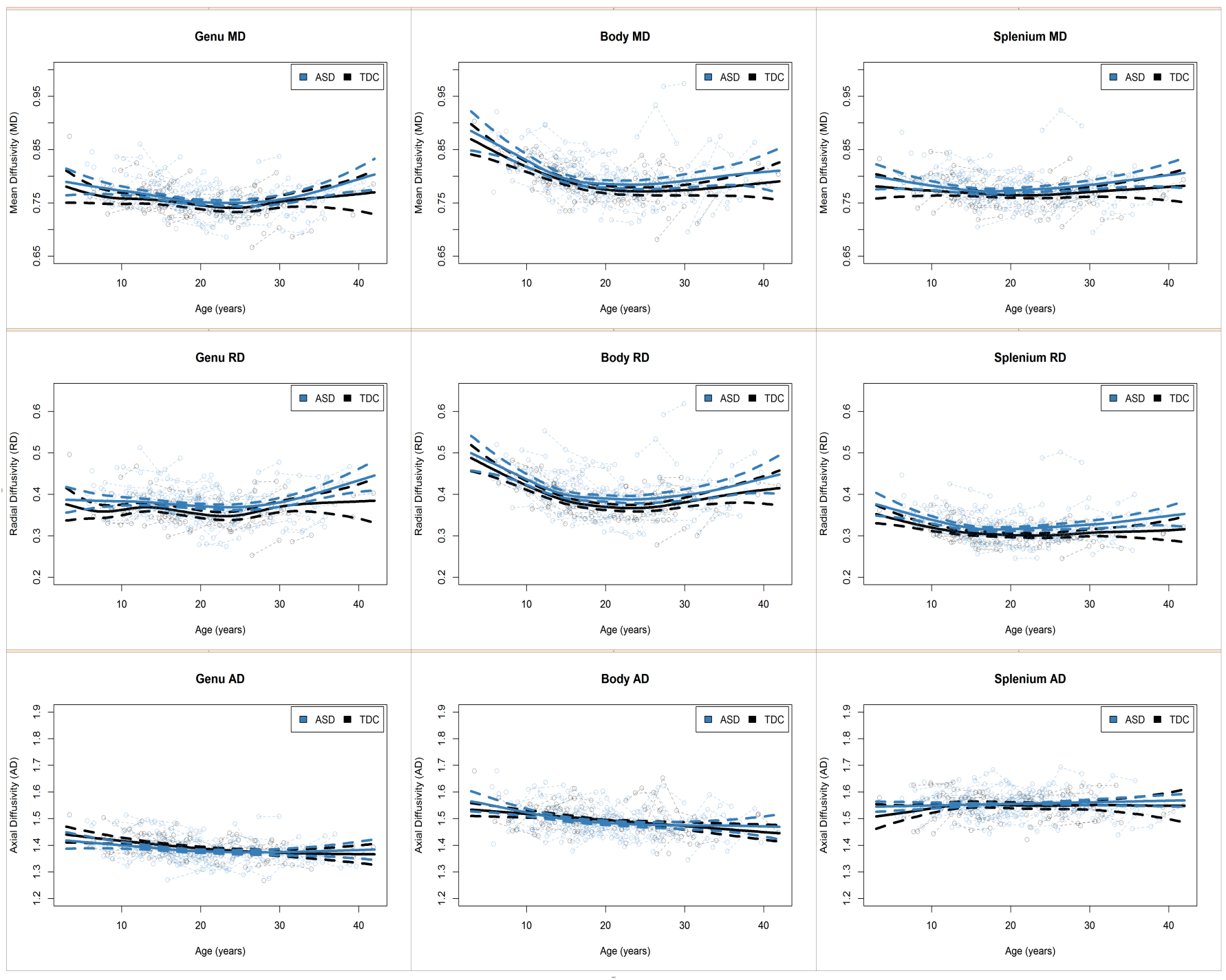
**Figure S4**. Generalized additive mixed model spline results for corpus callosum mean diffusivity (MD), radial diffusivity (RD), and axial diffusivity (AD) at Times 2, 3, and 4 (12-channel head coil).

**Figure S5**. Generalized additive mixed model spline results for fractional anisotropy (FA) of all time points, excluding scans where participants with ASD were on centrally active medications.


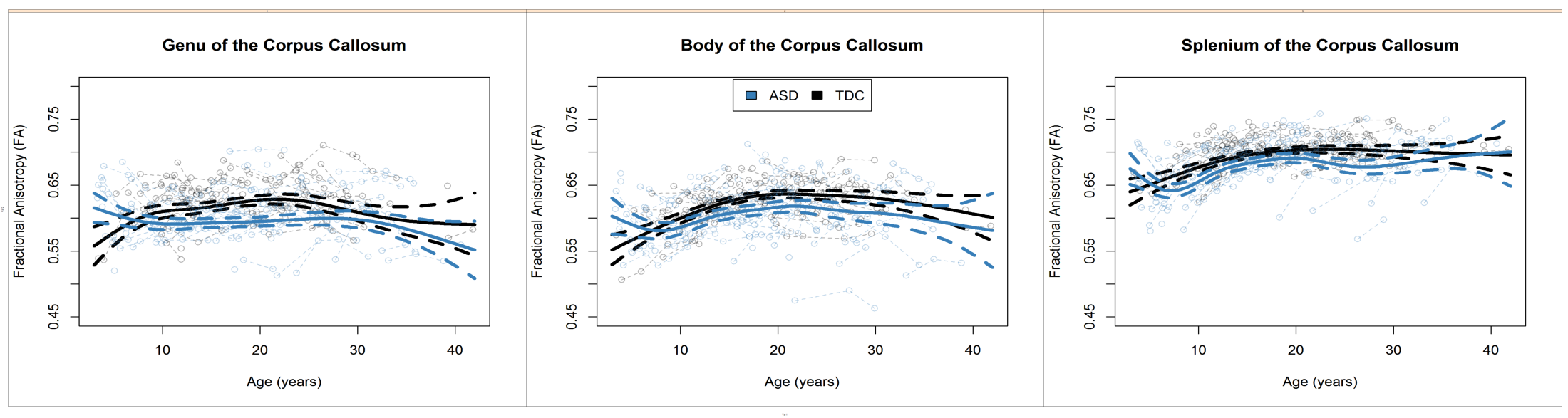


**Figure S6**. Generalized additive mixed model spline results for median diffusivity (MD), radial diffusivity (RD), and axial diffusivity (AD) of all time points, excluding scans where participants with ASD were on centrally active medications.


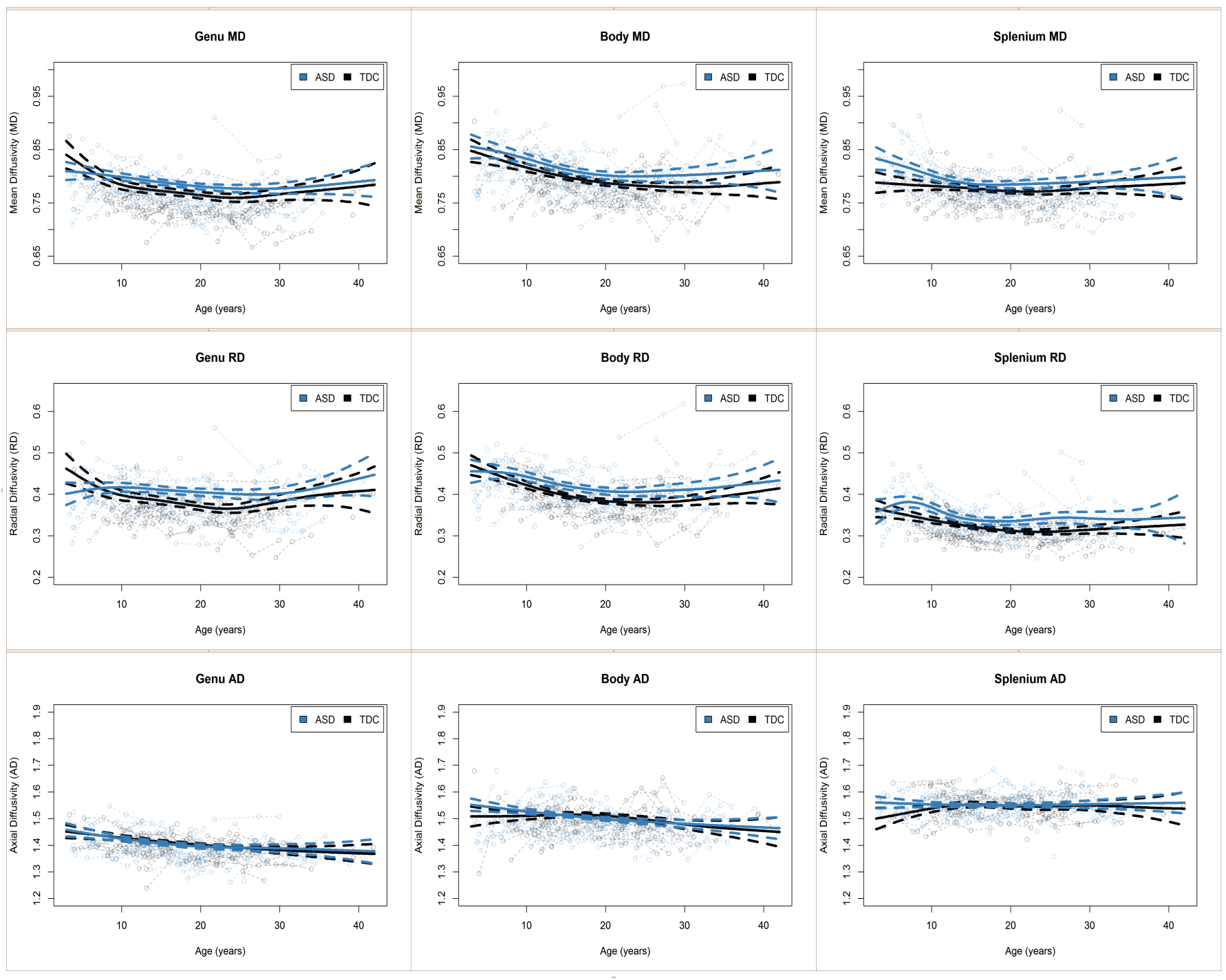
**Figure S7**. Generalized additive mixed model spline results for fractional anisotropy (FA) of all time points, excluding scans where participants with ASD were sedated.


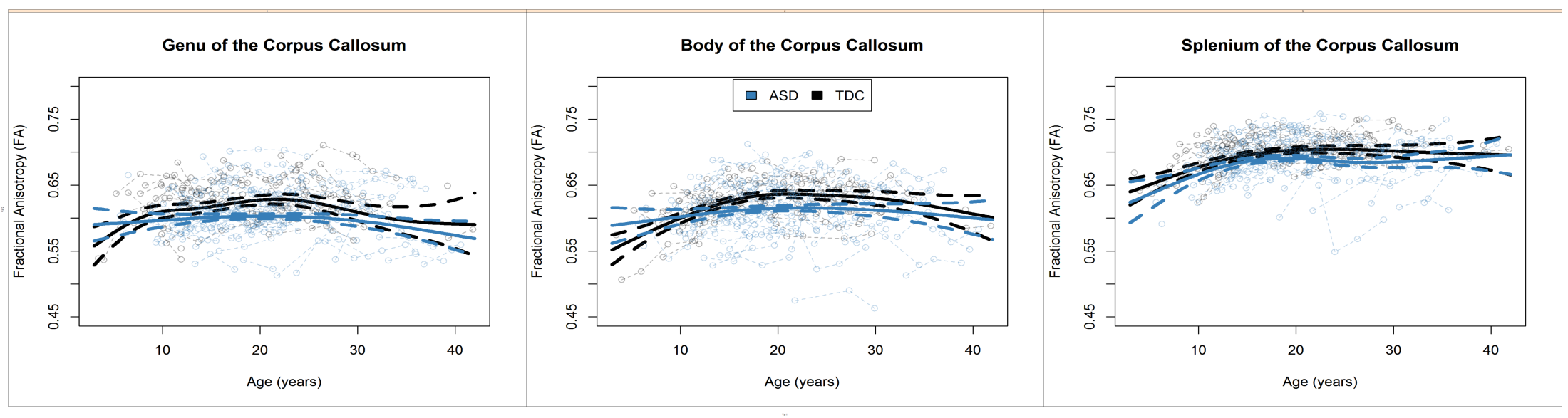


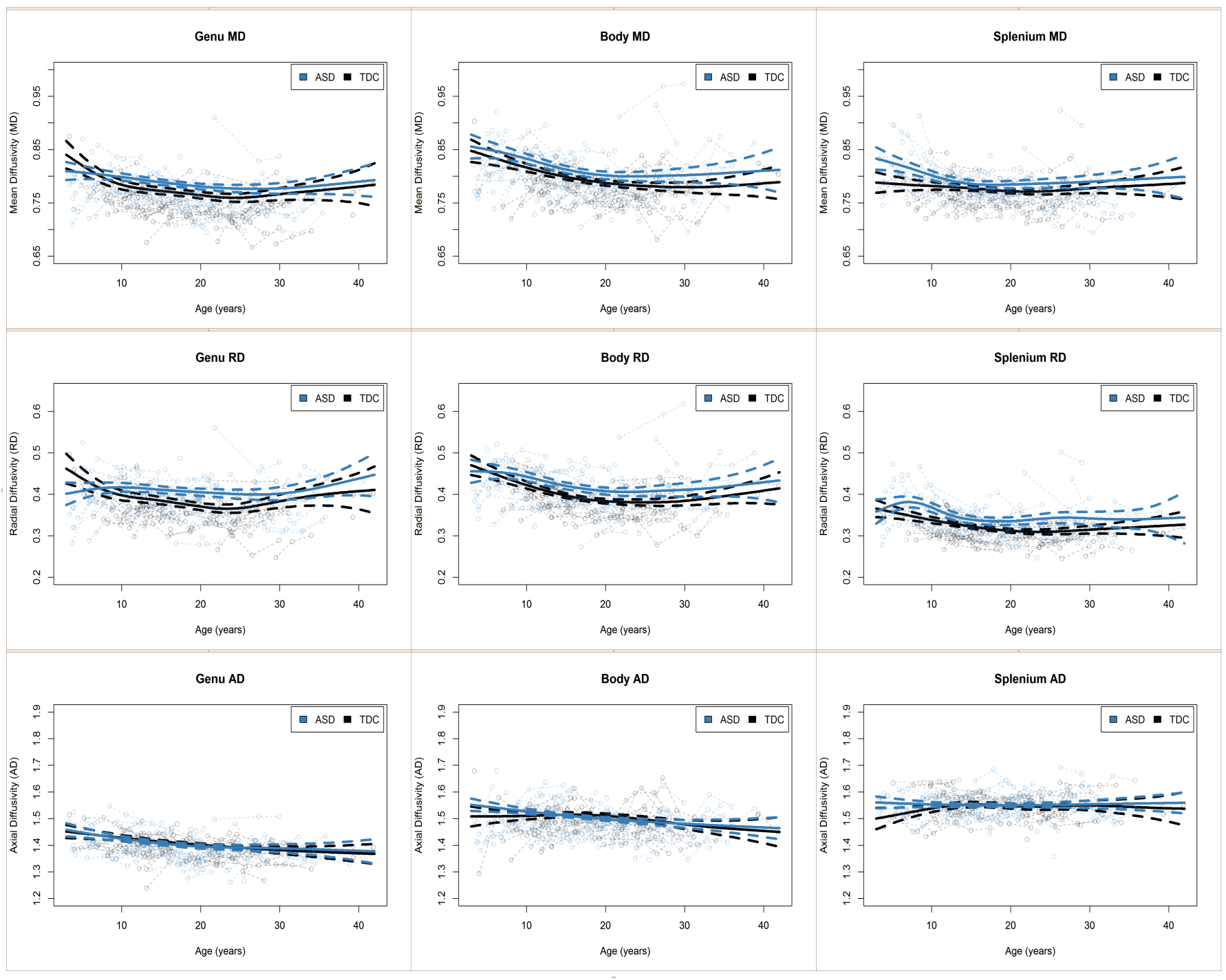
**Figure S8**. Generalized additive mixed model spline results for mean diffusivity (MD), radial diffusivity (RD), and axial diffusivity (AD) of all time points, excluding scans where participants with ASD were sedated.
